# Communicating the diagnosis of spinal muscular atrophy in endogamous vs. non-endogamous regions

**DOI:** 10.1186/s12883-024-03718-9

**Published:** 2024-07-26

**Authors:** Isabella Araujo Mota Fernandes, Renata Oliveira Almeida Menezes, Guilhermina Rego

**Affiliations:** 1 Faculty of Medicine, Lauro Wanderley University Hospital, University of Porto, Federal University of Paraíba, João Pessoa, Paraíba- Brazil, BR 230, Km 10 S /N Condomínio Villas do Atlântico casa 3 B. Intermares, Cabedelo, João Pessoa, CEP: 58102-202 Paraíba Brazil; 2 Federal University of Pernambuco and PhD in Legal and Social Sciences from the Federal University of Campina Grande, University of Rio Grande do Norte, Caicó, Brazil; 3 Faculty of Medicine, University of Porto, Porto, Portugal

**Keywords:** Endogamy, Autonomy, Communicating the diagnosis, Spinal muscular atrophy

## Abstract

**Introduction:**

The high prevalence of endogamy, or inbreeding, in northeastern Brazil, is due to historical and cultural factors, with large families living in cities far from the coast and subject to low socioeconomic and infrastructure levels. This breeding practice results in low genetic variability with an increased prevalence of rare autosomal recessive and neurodegenerative diseases, such as spinal muscular atrophy (SMA).

**Objective:**

Understanding the impact of communicating the diagnosis of SMA on the mental health of patients and their families and the differences between the Northeast (endogamous region) and the other regions of Brazil (non-endogamous ones).

**Methods:**

Cross-sectional study obtained through a structured questionnaire about the moment of receiving the SMA diagnosis, containing the Impact of Event Scale-Revised.

**Results and discussion:**

The sample consisted of 100 volunteers from all regions of Brazil, 47 patients diagnosed with SMA and 53 family members present at the time of the diagnosis. There was a predominance of females (83%) and homogeneity between the groups for the variables gender, age, color, education, religion, and SMA subtype (1, 2, 3, and 4). The Northeast region, representing 43% of the sample, despite being less economically favored, showed greater satisfaction with medical care and inclusion in health services, with less self-reported psychological trauma and fewer signs of post-traumatic stress disorder (PTSD) related to the moment of receiving the diagnosis. The non-endogamous regions, in turn, reported the presence of strong waves of emotion, sleep problems, feelings of irritability, anger, and the presence of bad thoughts related to this situation.

**Conclusion:**

The feeling of inclusion in health services and satisfaction with medical care in the endogamous region had a positive impact on the mental health of those involved, reducing psychological trauma and signs of PTSD arising from the communication of the SMA diagnosis.

**Supplementary Information:**

The online version contains supplementary material available at 10.1186/s12883-024-03718-9.

## Introduction

Endogamy was described in Brazil by Freire-Maia (1952/1957) when analyzing Catholic marriage records, a religion that represented 93% of the country in that period. The high frequency of endogamous marriages in the 19th century, which persisted into the 20th century, was justified by the low population density, lack of means of communication, isolation of small towns and villages, and large families with at least three children in large cities and four/five ones in rural areas [1]. Historical and cultural factors resulting from the influence of members from the Jewish community forcibly converted to Catholicism during the Inquisition (the New Christians), who migrated to the northeastern countryside, may have influenced the perpetuation of marriage practices among relatives, still observed nowadays [2, 3, 4]. Weller et al. (2012) found that, in the Northeast region, cities further away from the coast, with lower socioeconomic and infrastructure indices, have a higher frequency of endogamous marriages (6–41%), and, consequently, a higher prevalence of children with one or more physical, sensory, and/or mental disability, compared to non-endogamous ones [3].

The high prevalence of rare genetic diseases of neuromuscular etiology has been described in cities in northeastern Brazil, such as SPOAN in Serrinha dos Pintos-RN5; limb-girdle muscular dystrophy type R2 due to dysferlin deficiency in São Mamede-PB 6; Gaucher disease in Tabuleiro do Norte-CE; Mucopolysaccharidosis (MPS) types VI and VII in Monte Santo-BA; MPS type VII in Tucano and Araci-BA, Esperantina-PI, and in São José de Caiana-PB; among others [7]. The high rates of people with disabilities in endogamous populations are related to low genetic variability and consequently to the increase in autosomal recessive disorders, resulting in homozygous progeny for pathogenic variants that are rare in other regions [5}.

According to the Brazilian Institute of Geography and Statistics, in 2010, physical, sensory, and/or mental disabilities reached 23.9% of the Brazilian population, with higher prevalence in the Northeast region (26.6%) [8]. Santos et al. found that the genetic factor was responsible for 60% of the cases of disabilities in this region, while 30% had a secondary etiology, such as gestational and postpartum complications [9].

Given the above, the objective is to understand the impact of breaking bad news about the diagnosis of genetic and neurodegenerative diseases on the mental health of patients and their families, and to improve communication skills. Thus, spinal muscular atrophy (SMA), as it is a rare, genetic disease, with progressive functional loss of motor skills, that courses with limitations and disabilities, preserving cognition and having its onset predominantly in childhood, was selected as a prototype in the analysis of patients or family members who experienced the moment of breaking bad news about this diagnosis in the Northeast region (endogamous) and other regions of Brazil (non-endogamous).

The fact is that the genetic predetermination, which is more present in endogamous families, compared to non-endogamous families, means that the diagnosis of a rare, genetic and neurodegenerative disease results in repercussions on the mental health not only of patients, but of other family members. This research is justified by the gap in the literature regarding the breaking of bad news in neurology, negatively impacting the decision-making of patients, family members, and of the physicians responsible for it [10].

## Methodology

This research was approved by the ethics and research committee of the HULW-UFPB under opinion number 5,176,679 between September 2020 and March 2022. The sample collected throughout Brazil consisted of patients diagnosed with SMA or family members of patients with this disease who were present at the time of diagnosis. Their contacts were obtained through regional and national associations of patients with rare diseases. After being invited by telephone and giving their free and informed consent, the volunteers answered a structured questionnaire via *Google* forms. Only those with at least 18 years of age and without a history of cognitive impairment were included.

The study is cross-sectional and uses simple random sampling. The data collection instrument was developed for this research after reviewing the literature related to the theme [11–17] and contains quantitative, multiple-choice questions related to the moment of communicating the SMA diagnosis, in addition to the Impact of Event Scale-Revised (IES-R) to assess the presence of post-traumatic stress disorder (PTSD) related to the reporting of this diagnosis [18]. The structured questionnaire can be found in the supplementary file of this article.

Data were categorized and tabulated in a digital spreadsheet for further descriptive and inferential statistical analysis. The R software, version 4.1.1, was used and a significance of 5% was considered. The descriptive analysis was represented by measures of absolute and relative frequency, in addition to measures of central tendencies, such as mean and standard deviation. For inferential analysis, the Kolmogorov-Smirnov test, trial free version, was initially performed to prove the normality of the data. Subsequently, Fisher’s exact test and Pearson’s correlation test were performed [19].

## Results

**Fig. 1:**
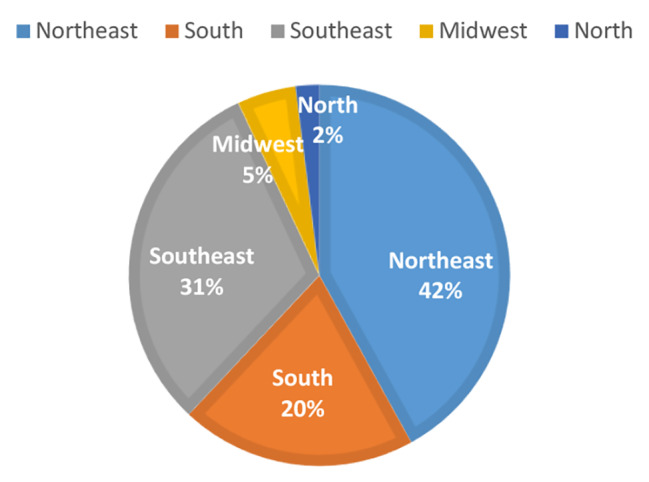
Percentage representation of endogamous region and of the non-endogamous region of the country

The sample was divided into an endogamous region (group 1), containing responses from the Northeast region, and a non-endogamous one (group 2), containing responses from other regions of the country (Fig. 1). There was no predominance of patients from specific medical services since the sample was collected from non-governmental associations of patients and family members.

Among the 100 volunteers who participated in the research, 53 were family members and 47 were patients diagnosed with SMA. 83% were female, aged 36.65 (± 10.19) years. The groups were homogeneous for the variables sex, age, color, education, religion, and SMA subtype (1, 2, 3, and 4), with higher family income in group 2 and higher frequency of history of genetic disease(s) in group 1 (Table 1). In the statistical evaluation, only yes or no answers related to the presence of genetic diseases in the family were considered, blank answers were not calculated. Regarding the SMA subtypes in the sample, 11% were type 1, 41% were type 2, 46% were type 3, and 2% were type 4. All responses related to patients with SMA type 1, the most severe subtype, were given by family members.

**Table 1:** Family income and family genetic history of volunteers in endogamous (Group 1) and non-endogamous (Group 2) regions

VARIABLES	Group 1		Group 2		*p*-value
	*N*	%	*N*	%	
**Family income**					
< 3 minimum wages	33	57.9%	24	42.1%	0.002*
Between 3-5minimum wages	6	26.1%	17	73.9%	
>= 6 minimum wages	3	15.8%	16	84.2%	
**Family genetic history**					
No	14	34.1%	27	65.9%	0.026*
Yes	10	55.6%	8	44.4%	

Pearson’s chi-square test; significance *p* < 0.05*.

The volunteers stated that they were evaluated by a varying number of physicians, in an appropriate environment, scheduled return appointments, understood the diagnosis, and received information with proportional quantity and quality between the groups (Table 2).

**Table 2:** Characteristics related to the moment of receiving the diagnosis of spinal muscular atrophy in endogamous (Group 1) and non-endogamous (Group 1) regions

VARIABLES	Group 1		Group 2		*p*-value
	N	%	N	%	
**Number of physicians seen until diagnosis**					
1–2	20	58.8%	14	41.2%	0.160
3–4	12	37.5%	20	62.5%	
5–6	5	35.7%	9	64,0.3%	
>=7	6	30,0.0%	14	70,0.0%	
**Did you feel included in the services you sought regarding the environment and the health team?**					
No	7	36.8%	12	63.2%	0.044*
Partially	12	31.6%	26	68.4%	
Yes	24	55.8%	19	44.2%	
**Degree of satisfaction with the medical care that preceded the definitive diagnosis**					
Dissatisfied	9	40.9%	13	59.1%	0.018*
Partially satisfied	2	12.5%	14	87.5%	
Satisfied	32	51.6%	30	48.4%	
**Adequacy of the diagnostic environment**					
No	1	25.0%	3	75.0%	0.412
Partially	5	31.3%	11	68.8%	
Yes	37	46.3%	43	53.8%	
**I have understood the diagnosis**					
No	3	42.9%	4	57.1%	0.966
Partially	11	42.3%	15	57.7%	
Yes	29	43.3%	38	56.7%	
**Scheduled follow-up visit**					
No	6	25.0%	18	75.0%	0.111
Cannot remember	4	57.1%	3	42.9%	
Yes	33	47.8%	36	52.2%	
**Classification of the quantity and quality of information about SMA provided by the health professional at the time of diagnosis**					
Dissatisfied	7	28.0%	18	72.0%	0.157
Partially satisfied	8	44.4%	10	55.6%	
Satisfied	28	41.1%	29	50.9%	
**Patient received genetic counseling**					
No	18	40.0%	36	60.0%	0.063
Cannot remember	1	50.0%	1	50.0%	
Yes	24	55,0.8%	19	44.0%	
**Psychological trauma due to the diagnostic process in the life of the patient and/or family**					
No	21	60.0%	14	40.0%	0.025*
Yes	13	29.5%	31	70.5%	
Perhaps	9	42.9%	12	57.1%	

Pearson’s chi-square test; significance *p* < 0.05*.

Over 90% of patients in both groups received the diagnosis in the presence of a companion. There was a trend, without statistical significance, of greater genetic counseling in the endogamous region, but this result is biased by the high prevalence of endogamy in the region. The volunteers in group 1 stated that they felt more pleased with the medical care they received, they felt included in the health services, and reported less psychological trauma related to the moment of receiving the SMA diagnosis (Table 2).

Signs of PTSD were present in 50% of the volunteers in both groups, while the absence of risk was greater in group 1, but without statistical significance (Table 3). Some signs related to PTSD were statistically significant, being more evident in group 2, such as the presence of strong waves of emotion (*p* = 0.026), sleep problems (*p* = 0.017), feeling irritable and angry (*p* = 0.029), and the presence of bad thoughts (*p* = 0.010) (Table 4).

**Table 3:** Post-traumatic stress after receiving the SMA diagnosis in endogamous and non-endogamous regions

VARIABLES	Group 1		Group 2		*p*-value
	*N*	%	*N*	%	
**Post-traumatic stress**					
Absence	29	50.9%	28	49.1%	0.168
Low risk	3	20.0%	12	80.0%	
Probable diagnosis	2	50.0%	2	50.0%	
PTSD	9	37.5%	15	62.5%	

Pearson’s chi-square test; significance *p* < 0.05*.

**Table 4:** Some signs related to PTSD with statistical significance when comparing endogamous and non-endogamous regions

VARIABLES		Group 1 *N* %		Group 2 *N* %		*p*-value
		*N* %		*N* %		
**Presence of strong waves of emotion**						
Extremely		1	11.1%	8	88.9%	0.026
Quite a bit		7	70%	3	30%	
Moderately		4	23.5%	13	76.5%	
A little bit		18	54.5%	15	44.5%	
Not at all		14	45.5%	17	55.4%	
**Sleep problems**						
Extremely		1	11.1%	8	88.9%	0.017
Quite a bit		6	75%	2	25%	
Moderately		3	25%	9	75%	
A little bit		24	55.8%	19	44.2%	
Not at all		10	35.7%	18	64.3%	
**Feeling irritable and angry**						
Extremely		1	14.3%	6	85.7%	0.029
Quite a bit		6	45.5%	5	54.5%	
Moderately		2	14.3%	12	85.7%	
A little bit		22	59.5%	15	40.5%	
Not at all		13	41.9%	18	59.1%	
**Presence of bad thoughts**						
Extremely		3	25%	9	75%	0.010
Quite a bit		8	50%	8	50%	
Moderately		2	11.8%	15	88.2%	
A little bit		14	70%	6	30%	
Not at all		17	48.6%	18	51.4%	

## Discussion

According to the literature, there is a higher prevalence of endogamous marriages in the Northeast region [1–9], consistent with the percentage of family history of genetic diseases in this study, confirming that endogamy rates remain high there, compared to other regions of Brazil (*p* = 0.026). This finding corroborates the division into group 1, or endogamous region, comprising the Northeast region; and group 2, or non-endogamous region, comprising the South, Southeast, Center-West, and North regions. The first has lower human development rates and per capita socioeconomic index, compared to group 2 [8], which is compatible with the percentage of patients who earn less than three minimum wages in group 1, and more than six minimum wages in group 2 (*p* = 0.002).

The lowest socioeconomic index in the Northeast region did not impact negatively the degree of satisfaction among its volunteers, who reported, in addition to greater satisfaction with the medical care received (*p* = 0.018), a greater feeling of inclusion in the health services in which they were cared for (*p* = 0.044). This region had even fewer reports of psychological trauma compared to how the SMA diagnosis was disclosed (*p* = 0.025). These findings are relevant and draw attention to the positive impact of the empathetic doctor-patient relationship and the welcome to patients provided by the health services when faced with the diagnosis of a progressive and disabling illness, such as SMA.

Despite the gap in research related to communicating the diagnosis of neuromuscular diseases, descriptions of failures in breaking the bad news regarding neurodegenerative diseases are related to the lack of technical training in neurologists and medical residents, generating moderate stress and feelings of anguish among those responsible for informing the patient [10]. Parents of children with neuromuscular diseases who were responsible for disclosing the diagnosis to them, without support from the attending physician, showed an increase in signs of PTSD, demonstrating the failure in the communication process between doctors, children, and legal guardians [20].

The current study showed that 50% of the sample had some sign of PTSD related to the moment of receiving the SMA diagnosis, regardless of region. However, there was a tendency for these signs in the non-endogamous region, but without statistical significance. This region showed the presence of strong waves of emotion, sleep problems, feelings of irritability and anger, and the presence of bad thoughts regarding this diagnosis. Individual susceptibility and the impact resulting from external factors, in this case, the diagnosis, may trigger chronic PTSD with varied signs, even silent suffering [21].

Barriers to communicating the diagnosis of neurodegenerative diseases occur when there is the absence of a companion and a return appointment already scheduled, by limited or unintelligible information, in addition to an environment without privacy [12]. In the current study, more than 90% of the patients received the diagnosis in the presence of a companion, 80% in an appropriate environment, 69% with a scheduled return appointment, and 67% reported understanding the diagnosis. These data confirm the improvement in diagnostic communication, as demonstrated by Anestis et al. (2020), despite dissatisfaction with the emotional support, the duration of the medical consultation, and the nature and quality of the information provided [22], represented by only 33% [19] of the participants, who felt included in the health services, and 75.4% (43), in group 2, who reported the presence or possibility of psychological trauma regarding the moment of receiving the SMA diagnosis.

The interaction of the physician, who is aware of the risks and benefits of decision-making; combined with clinical experience; and the SMA patient who experiences the progressive loss of his motor function, with fears, uncertainties, thoughts, and individualized interpretations of the reality of his culture, education, and financial situation affect different choices related to health [23]. Thus, the doctor-patient relationship can take on three different formats: shared decisions, informed decisions, and paternalism.

There will be cases in which patients are autonomous, and participate in all decisions (shared decisions) and others in which they will give their opinions and ask the doctor for the necessary measures according to the opinions offered (informed decisions) [24]. Some doctors limit the information under the justification of sparing the patient, establishing behaviors that ignore their will and individual needs, and seriously compromising their autonomy in decision-making (paternalism) [23]. However, some patients entrust all therapeutic decisions to their physician, so that they act in agreement with science and their experience, spontaneously limiting their decision-making capacity, which is also a paternalistic posture [25].

It is fundamental to understand who receives the bad news so that the communication is centered on the interlocutor. When it comes to analyzing autonomy, the model by which decisions will be taken stands out [23]. Bearing in mind that each patient reacts differently to the same disease and the same treatment, knowing the individuality of each of them, making them feel included in the health service, and satisfied with the medical care will help them in making a decision. Poor choices or the poor perception of the model to be used may be the result of protocols that do not assess the individual as such [25], or of poor communication [26] which, in addition to not clarifying the diagnosis, may generate traumatic sensations and signs of post-traumatic stress that influence freedom of choice and consent, and both are essential in autonomous decisions [10, 20].

Given the above, the endogamous and non-endogamous regions were similar in terms of the fact that the SMA diagnosis was communicated by physicians, and their participation was required in both regions, in the presence of a companion, and an appropriate environment. Most volunteers understood the diagnosis and most of them reported being satisfied, or partially satisfied, with the amount and quality of the information provided, in addition to having a scheduled return appointment. Although there was no statistical significance, there was a trend towards a higher frequency of genetic counseling in the Northeast region. However, the high prevalence of endogamy may be a bias factor.

It is concluded, therefore, that feeling included in health services and satisfied with medical care, in the endogamous region, had a positive impact on the short and long-term mental health of patients and their families, with a reduction in psychological trauma and signs of PTSD related to receiving the SMA diagnosis.

The expansion of studies related to communicating the diagnosis of rare neuromuscular diseases, to understand which factors positively impact this process, as well as which barriers compromise the degree of satisfaction and the feeling of inclusion in health services, is relevant for improving communication techniques and, consequently, obtaining well-informed decision-making, free of psychological trauma in the short and long term, respecting the autonomy of those involved [26].

## Study limitations

Among the limitations of this study, a single neuromuscular disease was evaluated, SMA, and there may be differences when evaluating different diseases. Other endogamous regions should be investigated to confirm if the data are replicable or if they only represent the studied population.

### Electronic supplementary material

Below is the link to the electronic supplementary material.

Supplementary Material 1

## Data Availability

The datasets used and/or analysed during the current study are available from the corresponding author on reasonable request.
