# Supplementary material for: Communicating the diagnosis of spinal muscular atrophy in endogamous vs. non-endogamous regions
Source: BMC Neurol. 2024 Jul 26;24:261. doi: 10.1186/s12883-024-03718-9 (PMC11282695; doi:10.1186/s12883-024-03718-9)
Supplement: Supplementary file 1 — Supplementary Material 1 [file 12883_2024_3718_MOESM1_ESM.pdf]

Structured interview

## Social and Epidemiological Profile

1. Sex

   ( ) Male ( ) Female ( ) I do not wish to inform

2. Age (in years, put only the number): ______

3. Color

   ( ) Yellow ( ) White ( ) Black ( ) Brown ( )Other

4. Education

   ( ) Illiterate ( ) Complete primary education ( ) Complete high school ( ) Incomplete high school ( ) University education ( ) Postgraduate studies

5. Region from Brazil

   ( ) Center-west ( ) North East ( ) North ( ) South East ( ) South

6. Family income

   ( ) < 1 minimum wage ( ) >= 6 minimum wages ( ) between 1-2 minimum wages ( ) between 3-5 minimum wages

7. Degree of kinship with the SMA patient

   ( ) Father ( ) Mother ( ) Uncle/Aunt ( ) Sister/Brother ( ) Grandfather/ Grandmother ( ) Other

8. Did you participate to the investigation process of the diagnosis of Spinal Muscular Atrophy?

   ( ) Yes ( ) No ( ) Partially

9. Definitive diagnosis

   ( ) SMA type 0 ( ) SMA type 1 ( ) SMA type 2 ( ) SMA type 3 ( ) SMA type 4

## The path to diagnosis

10. How long between the investigation of the first symptoms and the diagnostic conclusion?

    ( ) < or = 1 year, ( ) between 1 and 2 years, ( ) between 2 and 4 years, ( ) between 4 and 6 years and ( ) > 6 years

11. Number of physicians seen until diagnosis

    1-2 ( ), 3-4 ( ), 5-6 ( ), >ou = 7 ( )

12. Degree of satisfaction with the medical care that preceded the definitive diagnosis

    Dissatisfied ( ) Partially satisfied ( ) Satisfied ( )

13. Was the diagnosis environment adequate?

    No ( ) Partially ( ) Yes ( )

14. Did you understand the diagnosis?

    No ( ) Partially ( ) Yes ( )

15. Was a follow-up appointment scheduled?

    No ( ) Cannot remember ( ) Yes ( )

16. Classification of the quantity and quality of information about SMA provided by the health professional at the time of diagnosis

    Dissatisfied ( ) Partially satisfied( ) Satisfied ( )

17. Was genetic counseling performed with the patient?

    No ( ) Cannot remember ( ) Yes ( )

18. Did you feel included in the health services sought in relation to the environment and health professionals?

    ( ) Yes ( ) No ( ) Partially

**Perceptions of post-diagnosis family members and patients**

19. Did the investigation trajectory up to the time of diagnosis leave any psychological trauma in the lives of patients and/or their families?

    ( ) Yes ( ) No ( ) Perhaps

**Impact of Event Scale - Revised (IES-R)**

Instructions: Below is a list of difficulties people sometimes have after stressful life events. Please read each item, and then indicate how distressing each difficulty has been for you DURING THE PAST SEVEN DAYS with respect to the communication of SMA diagnosis. How much were you distressed or bothered by these difficulties?

20. Any reminder brought back feelings about it

( ) Not at all ( )A little bit ( )Moderately ( )Quite a bit ( )Extremely

21. I had trouble staying asleep

( ) Not at all ( )A little bit ( )Moderately ( )Quite a bit ( )Extremely

22. Other things kept making me think about it

( ) Not at all ( )A little bit ( )Moderately ( )Quite a bit ( )Extremely

23. I felt irritable and angry

( ) Not at all ( )A little bit ( )Moderately ( )Quite a bit ( )Extremely

24. I avoided letting myself get upset when I thought about it or was reminded of it

( ) Not at all ( )A little bit ( )Moderately ( )Quite a bit ( )Extremely

25. I thought about it when I didn't mean to

( ) Not at all ( )A little bit ( )Moderately ( )Quite a bit ( )Extremely

26. I felt as if it hadn't happened or wasn't real

( ) Not at all ( )A little bit ( )Moderately ( )Quite a bit ( )Extremely

27. I stayed away from reminders about it

( ) Not at all ( )A little bit ( )Moderately ( )Quite a bit ( )Extremely

28. Pictures about it popped into my mind

( ) Not at all ( )A little bit ( )Moderately ( )Quite a bit ( )Extremely

29. I was jumpy and easily startled

( ) Not at all ( )A little bit ( )Moderately ( )Quite a bit ( )Extremely

30. I tried not to think about it

( ) Not at all ( )A little bit ( )Moderately ( )Quite a bit ( )Extremely

31. I was aware that I still had a lot of feelings about it, but I didn't deal with them

( ) Not at all ( )A little bit ( )Moderately ( )Quite a bit ( )Extremely

32. My feelings about it were kind of numb

( ) Not at all ( )A little bit ( )Moderately ( )Quite a bit ( )Extremely

33. I found myself acting or feeling as though I was back at that time

( ) Not at all ( )A little bit ( )Moderately ( )Quite a bit ( )Extremely

34. I had trouble falling asleep

( ) Not at all ( )A little bit ( )Moderately ( )Quite a bit ( )Extremely

35. I had waves of strong feelings about it

( ) Not at all ( )A little bit ( )Moderately ( )Quite a bit ( )Extremely

36. I tried to remove it from my memory

( ) Not at all ( )A little bit ( )Moderately ( )Quite a bit ( )Extremely

37. I had trouble concentrating

( ) Not at all ( )A little bit ( )Moderately ( )Quite a bit ( )Extremely

38. Reminders of it caused me to have physical reactions, such as sweating, trouble breathing, nausea, or a pounding heart

( ) Not at all ( )A little bit ( )Moderately ( )Quite a bit ( )Extremely

39. I had dreams about it

( ) Not at all ( )A little bit ( )Moderately ( )Quite a bit ( )Extremely

40. I felt watchful or on-guard

( ) Not at all ( )A little bit ( )Moderately ( )Quite a bit ( )Extremely

41. I tried not to talk about it

( ) Not at all ( )A little bit ( )Moderately ( )Quite a bit ( )Extremely
